# Supplementary material for: An integrated leaf trait analysis of two Paleogene leaf floras
Source: PeerJ. 2023 Apr 10;11:e15140. doi: 10.7717/peerj.15140 (PMC10100813; doi:10.7717/peerj.15140)
Supplement: Supplemental Information 1 — Additional information regarding the geological background, fossil site features, and collection history. [file peerj-11-15140-s001.pdf]

Supplemental Article S1

Detailed information regarding the geological background, fossil site features, and collection history are available here to supplement the section of the article.

Geological background and fossil site features

Volcanic activity during the Paleogene to Neogene in Northern Bohemia (Czech Republic) and the Upper Lusatia in southeast Saxony (Germany) was caused by tectonic movements in the ENE–WSW trending Ohře (Eger) Graben, the eastern part of the European Cenozoic rift system (*Ziegler, 1992*; *Ziegler, 1994*). The Ohře Graben is characterized by volcanic complexes, wedged brown coal basins, and by the formation of marginal depressions (*Akhmetiev, Walther & Kvaček, 2009*). During the early Oligocene, such a local depression with a paleo-lake was formed between the localities of Seifhennersdorf (Saxony, Germany) and Varnsdorf (Ústí nad Labem Region, Czech Republic). The lake infilling is recorded by a up to 60 meters thick volcano-sedimentary sequence that contains interbeds of fossiliferous diatomite seams (*Ahrens, 1959*; *Walther & Kvaček, 2007*). The sequence ends with a potassium-argon-dated basaltic lava flow of 30.7 ± 0.7 million years (*Bellon et al., 1998*). Overall, five seams of lacustrine diatomite developed in temperate humid but seasonal paleoclimate (*Schiller, 2007*). Seams four and five, in the lowest part of the sequence, reached the highest thicknesses (four and 10 meters) and contained rich fossil fauna and the Seifhennersdorf flora (*Walther & Kvaček, 2007*). Toward the top of the sequence, volcanic activity increased, resulting in thinner diatomite seams. A thin coal seam was deposited above the last diatomite seam at the top of the sequence, representing the lake evolution's final stage. It is assumed that the Seifhennersdorf lake persisted only for a relatively short period (~ 100.000 years) till it was filled with sediments and finally covered by the lava flow (*Ahrens, 1959*).

Volcanism and tectonic movements controlled the lake's evolution and shaped the morphology of the lake's surroundings. That caused the development of various habitats inhabited by diverse plant communities at the shore of the Seifhennersdorf lake and within its proximity (*Walther & Kvaček, 2007*). Mixed mesophytic forest with, e.g., *Acer*, *Betula*, *Carpinus*, *Carya*, *Daphnogene*, *Dombeyopsis*, *Laurophyllum*, *Platanus, Rosa*, *Sloanea*, *Ulmus*, and *Zelkova* developed on hilly non-waterlogged sites whose soils derived from deeply weathered Lausitz Granodiorite. Flat and periodically flooded lake shores favored riparian forest development with, e.g., *Alnus*, *Carpinus*, *Carya*, *Eotrigonobalanus,* and *Populus* on fertile soils of alkaline volcanic rocks. Local rather permanently flooded depressions favored the colonization by a *Taxodium*-dominated swamp forest (*Walther & Kvaček, 2007*).

Besides the fossil flora, faunal elements are preserved in diatomite seams four and five. Fishes (*Böhme, 2007a*), amphibians (*Böhme, 2007b*), and several fossil insect species (*Prokop & Fikáček, 2007*) were described from Seifhennersdorf. Thirteen families of seven insect orders, represented by body or trace fossils, encompass the entomofauna (*Tietz, Berner & Mätting, 1998*; *Prokop & Fikáček, 2007*). Its composition resembles the entomofauna from the early Oligocene Czech site Kundratice (*Prokop, 2003*). The Seifhennersdorf insect fossil record is dominated by terrestrial insects, mainly beetles (Curculionidae). Aquatic species like caddis flies (mainly larval cases) and

water beetles are only present as accessories (*Fikáček, Hájek & Prokop, 2008*). These aquatic insects imply the presence of a distinct littoral zone with rich submerged plant species and riparian vegetation, providing potential oviposition sites for semiaquatic insects. This implication follows the above-mentioned palaeobotanical reconstructions (*Walther & Kvaček, 2007*). Oviposition marks of a fossil damselfly (?Lestidae) on leaves of *Carpinus grandis* (Betulaceae) described by *Hellmund & Hellmund (1996)* represent the first study of insect damage types from Seifhennersdorf. The first extensive but not published analysis of insect damage types was conducted by *Koch (2011)*. The analysis is revised and extended in the present study.

The fossil site of Berand near the village of Suletice, called Suletice-Berand, is situated in the east of Ústí nad Labem in Northern Bohemia (Czech Republic) within the České středohoří Mountain's volcano-sedimentary complex (ČsMts. complex). The ČsMts. Complex, as a geological unit of the Ohře Graben, is characterized by a lithostratigraphy showing a polygenetic assemblage of alkaline superficial and intrusive volcanism, pyroclastics, and accompanying sedimentary intercalations (*Cajz, 2000*; *Akhmetiev, Walther & Kvaček, 2009*). The fossil flora of Suletice-Berand is preserved in freshwater diatomite embedded in a sequence of basaltoid pyroclastics of the Ústí Formation (*Cajz, 2000*). Contrary to Seifhennersdorf, knowledge about local geology, stratigraphic position, and the structure of the paleo-lake, most probably the infilling of a volcano-tectonic depression, are limited and only partly clarified by studies of core drillings and in test pits (*Kvaček & Walther, 1995*; *Walther, 2004*). Paleobotanical investigations indicate the lack of swampy and riparian habitats like in Seifhennersdorf. Non-waterlogged areas were inhabited by a mixed mesophytic forest characterized by fossil-species of, e.g., *Acer*, *Daphnogene, Engelhardia*, *Laurophyllum, Leguminosites*, *Platanus*, *Sloanea,* and *Zelkova* (*Kvaček & Walther, 1995*; *Akhmetiev, Walther & Kvaček, 2009*).

According to their embedding in volcano-sedimentary sequences (e.g., infillings of volcano-tectonic depressions, maar, or crater lakes), fossil floras, like Seifhennersdorf and Suletice-Berand, have been denominated as 'volcanic floras' (*Walther, 2004*; *Akhmetiev, Walther & Kvaček (2009*). This kind of vegetation is controlled by volcanic activity and tectonics that cause a heterogeneous landscape with uplands and depressions and, thus, diverse microclimate conditions. Additionally, the edaphic factors of volcanogenic paleosoils might have been essential for the vegetation composition (*Kvaček & Walther, 2001*). However, it could not be demonstrated that fossil vegetation colonized such paleo-soils. Compared to leaf assemblages from central and eastern German Paleogene sites of the coastal lowland plains, these sites provide a higher diversity in fossil-species (e.g., *Kunzmann & Walther, 2012*; *Kunzmann et al., 2016*) and consist predominantly of zonal (hinterland) vegetation elements (*Kvaček & Walther, 2001*). Based on the Integrated Plant Record Vegetation Analysis, the floras of Seifhennersdorf and Suletice-Berand are assigned to a vegetation type defined as an ecotone between broad-leaved evergreen forest and mixed mesophytic forest (*Teodoridis & Kvaček, 2015*).

Collections – history and collecting process

As mentioned above, the specimens in the Seifhennersdorf collection come from a localized area between Seifhennersdorf and Varnsdorf. The small lagerstätte of coal and lacustrine diatomite was excavated in two mining periods. Between 1837 and 1856, small coal deposits were mined near the surface. After World War II, from 1951 to 1957, the Geological State Office of Saxony reopened the pit as an exploratory mine for resources (e.g., *Walther, 1996*; *Walther & Kvaček, 2007*). Seifhennersdorf specimens were first collected and described by H. Engelhardt in the late 1860ies (*Engelhardt, 1870*) from dumps of a mine that excavated the small near-surface lignite seam and underlying first diatomite. In the 1950s – 1990s, several paleobotanists and private collectors collected the most significant part of the Seifhennersdorf collection from the mining dump of an exploitation mine (*Walther, 1964*; *Walther, 1984*). The government of the former German Democratic Republic (GDR) kept geological prospecting of resources secret so that publications of geological data, theses, and direct sampling in the underground mine were restricted (*Walther, 1996*). The later accessibility of unpublished reports and theses revealed essential details about the geological background. Most of the fossil material excavated by the exploratory mine and deposited in dumps coming from the deeper and thicker diatomite seams four and five. Seams 2 and 3 of the section are thin and without fossils (*Ahrens, 1959*; *Walther & Kvaček, 2007*). Since no significant floristic difference between the diatomite layers was detected, we consider the collected Seifhennersdorf specimens as a unit (*Walther & Kvaček, 2007*). According to catalog numbers, the Seifhennersdorf collection at the Senckenberg Natural History Collections Dresden, Museum of Mineralogy and Geology (MMG) includes >5000 hand-sized specimens with fossil plant remains. Since most of the collection was obtained through intensive and systematic sampling (including fragments) of mining dumps during 1951-1994, we assume that sampling biases did not strongly influence the diversity of leaf morphotypes.

Suletice-Berand specimens were collected from a few cubic meters of rocks from a well shaft during a single fieldwork campaign in 1896 by the paleobotanists J. Deichmüller and P. Menzel (*Engelhardt, 1898*; *Kvaček & Walther, 1995*). The differently hand-sized specimens and leaf fragments suggest that the collectors took as much material as possible and did not pre-select better-preserved fossils in the field. According to catalog numbers, the Suletice-Berand collection at the MMG includes 918 hand-sized specimens with fossil plant remains representing all material from the well shaft.

In summary, we assume that specimen sampling generating the two historical collections was conducted to capture the fossil-species diversity of the sites and was not focused on insect damage types or leaf specimen attractiveness.

References

Ahrens, H. 1959. Stratigrafisch-tektonische Untersuchung im Tertiär von Seifhennersdorf. *Geologie* 8: 340-341.

Akhmetiev M, Walther H, Kvaček Z. 2009. Mid-latitude Palaeogene floras of Eurasia bound to volcanic settings and palaeoclimatic events – experience obtained from the Far East of Russia (Sikhote-Alin´) and Central Europe (Bohemian Massif). *Fossil Imprint/ Acta Musei Nationalis Pragae, Series B – Historia Naturalis* 65:61–129.

Bellon H, Bůžek Č, Gaudant J, Kvaček Z, Walter H. 1998. The Česke Středohoří magmatic complex in Northern Bohemia 40K-40Ar ages for volcanism and biostratigraphy of the Cenozoic freshwater formations. *Newsletters on Stratigraphy* 36:77–103 DOI 10.1127/nos/36/1998/77.

Böhme M. 2007a. Revision of the cyprinids from the Early Oligocene of the České Středohoří Mountains, and the phylogenetic relationships of *Protothymallus* Laube 1901 (Teleostei, Cyprinidae, Gobioninae). *Fossil Imprint/ Acta Musei Nationalis Pragae, Series B – Historia Naturalis* 63:175–194.

Böhme M. 2007b. The frog from Seifhennersdorf. *Fossil Imprint/ Acta Musei Nationalis Pragae, Series B – Historia Naturalis* 63:215.

Cajz V. 2000. Proposal of lithostratigraphy for the České středohoří Mts. volcanics. *Bulletin of Geosciences* 75:7–16.

Engelhardt H. 1870. Flora der Braunkohlenformation im Königreich Sachsen. *Preisschriften gekrönt und herausgegeben von der Fürstlich Jablonowski'schen Gesellschaft zu Leipzig* 16:1–69.

Engelhardt H. 1898. Die Tertiaerflora von Berand im Böhmischen Mittelgebirge. Ein neuer Beitrag zur Kenntnis der fossilen Pflanzen Böhmens. *Abhandlungen des deutschen naturwissenschaftlich-medizinischen Vereines für Böhmen "Lotos"* 1:75–123.

Fikáček M, Hájek J, Prokop J. 2008. New records of the water beetles (Coleoptera: Dytiscidae, Hydrophilidae) from the central European Oligocene-Miocene deposits, with a confirmation of the generic attribution of *Hydrobiomorpha enspelense* Wedmann 2000. *Annales de la Societe Entomologique de France* 44:187–199 DOI 10.1080/00379271.2008.10697555.

Hellmund M, Hellmund W. 1996. Zur endophytischen Eiablage fossiler Kleinlibellen (Insecta, Odonata, Zygoptera) mit Beschreibung eines neuen Gelegetyps. (Endophytic reproduction of fossil damselflies (Insecta, Odonata, Zygoptera), with the description of a new type of egg-sets.) *Mitteilungen der Bayerischen Staatssammlung für Paläontologie und Historische Geologie* 36:107–115.

Koch M. 2011. Plant-insect interaction and the climate implications in the Early Oligocene of Seifhennersdorf (Eastern Germany) and Kundratice (Southern Czech Republic). Diploma Thesis. University of Bonn.

Kunzmann L, Walther H. 2012. Early Oligocene plant taphocoenoses of the Haselbach megafloral complex and the reconstruction of palaeovegetation. *Palaeobiodiversity and Palaeoenvironments* 92:295–307 DOI 10.1007/s12549-012-0078-4.

Kunzmann L, Kvaček Z, Teodoridis V, Müller C, Moraweck K. 2016. Vegetation dynamics of riparian forest in central Europe during the late Eocene. *Palaeontographica Abteilung B* 295:69–89 DOI 10.1127/palb/295/2016/69.

Kvaček Z, Walther H. 1995. The Oligocene volcanic flora of Suletice-Berand near Ústí nad Labem, North Bohemia–a review. *Fossil Imprint/Acta Musei Nationalis Pragae, Series B – Historia Naturalis Praha* 50:25–54 DOI 10.2478/if-2018-0018.

Kvaček Z, Walther H. 2001. The Oligocene of Central Europe and the develoment of forest vegetation in space and time based on megafossils. *Palaeontographica Abteilung B* 259:125–148.

Prokop J. 2003. Remarks on palaeoenviromental changes based on reviewed Tertiary insect associations from the Krušné hory piedmont basins and the České středohoří Mts. in Northwestern Bohemia (Czech Republic). *Acta Zoologica Cracoviensia* 46:329–344.

Prokop J, Fikáček M. 2007. Early Oligocene insect fauna from Seifhennersdorf (Saxony, Germany). *Fossil Imprint/ Acta Musei Nationalis Pragae, Series B – Historia Naturalis* 63:205–213.

Schiller W. 2007. Siliceous microfossils from the Oligocene tripoli-deposit of Seifhennersdorf. *Fossil Imprint/ Acta Musei Nationalis Pragae, Series B – Historia Naturalis* 63:195–204.

Teodoridis V, Kvaček Z. 2015. Palaeoenvironmental evaluation of Cainozoic plant assemblages from the Bohemian Massif (Czech Republic) and adjacent Germany. *Bulletin of Geosciences* 90:695–720 DOI 10.3140/bull.geosci.1553.

Tietz O, Berner T, Mätting E. 1998. Insekten aus dem Unteroligozän von Seifhennersdorf in der Oberlausitz. *Abhandlungen und Berichte des Naturkundemuseums Görlitz* 70:139–154.

Walther H. 1984. Paläobotanische Sammlungstätigkeit im Tertiär der Oberlausitz (1866-1983). *Abhandlungen und Berichte des Naturkundemuseums Görlitz* 58:23–32.

Walther H. 1996. Das Tertiär-Vorkommen von Seifhennersdorf (Oberlausitz, Deutschland). *Neues Jahrbuch für Geologie und Paläontologie-Abhandlungen* 200:5–26.

Walther H, Kvaček Z. 2007. Early Oligocene flora of Seifhennersdorf (Saxony). *Fossil Imprint/Acta Musei Nationalis Pragae, Series B–Historia Naturalis* 63:85–174.

Ziegler PA. 1992. European Cenozoic rift system. *Tectonophysics* 208:91–111 DOI 10.1016/0040-1951(92)90338-7.

Ziegler PA. 1994. Cenozoic rift system of western and central Europe: an overview. *Geologie en Mijnbouw* 73:99–127.
