# Supplementary material for: An integrated leaf trait analysis of two Paleogene leaf floras
Source: PeerJ. 2023 Apr 10;11:e15140. doi: 10.7717/peerj.15140 (PMC10100813; doi:10.7717/peerj.15140)
Supplement: Supplemental Information 2 — The results of multivariate analyses performed on Dataset 4 without LMA imputation (n = 228 specimens; see Material & Methods) are shortly presented. [file peerj-11-15140-s002.pdf]

Supplemental Article S2

Results of the multivariate analyses without imputation of LM_A.

The results of multivariate analyses performed on Dataset 4 without LM_A imputation (n = 228 specimens; see Material & Methods) are shortly presented.

Principal Component Analysis

The two first axes, namely PC1 and PC2, explain 85.0 % of the variance (56.1 % and 29.0 %, respectively). PCA results obtained on the smaller Dataset 4 (i.e., with specimens having their petioles preserved and thus an LM_A estimation) are fairly consistent with the results obtained from the larger Dataset 5 (i.e., containing also simulated LM_A values), with similar contributions of the different quantitative variables to specimen ordination (Table 1). Note, however, that unlike the results shown in the paper's core, the PC1 axis is slightly more affected by LM_A values than leaf length (Figs. 1A and 1C).

Generalized Linear Models

The best models to explain LM_A, leaf size, and plant-insect interaction variations within the data (Dataset 4, n = 228) are listed below:

LM_A ~ Leaf size + Phenology + Species (+ Locality) (SI-M1)

Leaf size ~ Species + IA + Phenology (SI-M2)

The variables best explain LM_A variations are leaf size, plant phenology (obtained from modern relative's ecological preferences), and species (Table 2). LM_A is negatively affected by leaf size but positively associated with evergreen and, to a lesser extent, semi-deciduous fossil-species (as visible in Figures 1A and 1E). The correlation between LM_A and phenology suggests that the assumption of unchanged phenology between the fossil-species and their modern relatives is likely valid at the assemblage level. Similarly, leaf size variations are also partly explained by *species* and *phenology*, with an additional effect of preservation (Area Index, IA; Table 2). Negative relationships are observed between leaf size, leaf preservation (IA), and leaf life span (i.e., the smallest leaves are the best preserved and are related to evergreen species).

GLM analyses provided no significant model to explain plant-insect interaction with vegetation properties based on Dataset 4 (Table 2). First, no binomial model was found to explain the presence/absence of herbivory. Thus, it is impossible with the data to tell whether some leaf traits (leaf area, LM_A, or TCTs), phenology, or assemblage properties (locality, taxonomic composition, or leaf preservation index) may affect the probability of a leaf being damaged. Second, the models using the damaged leaves only (n = 18), with the damaged area (in mm^2) or herbivory index as response variables, either didn't converge or showed no relationships with other variables.

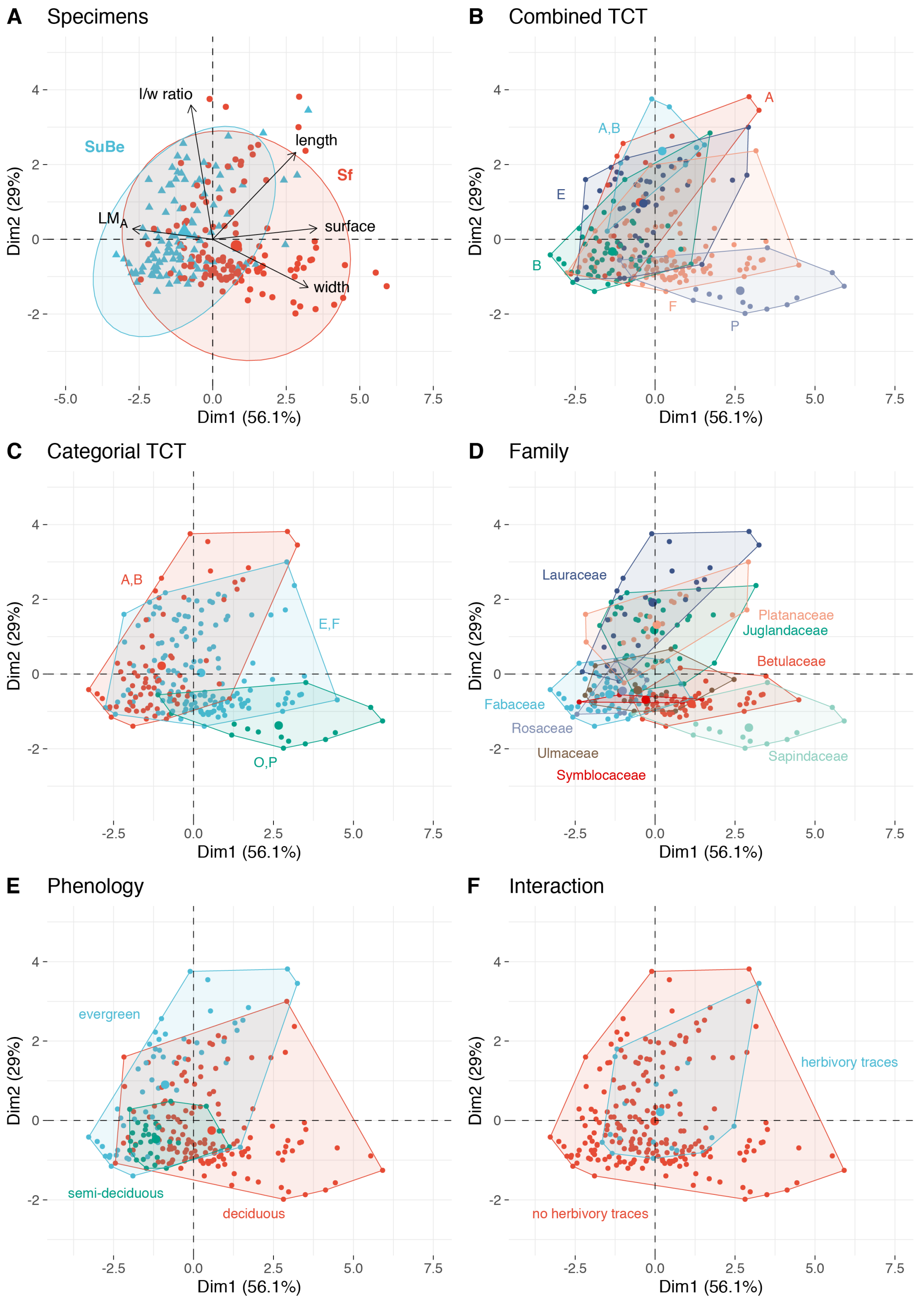

Figure 1 The morphospace of leaves is built from the two first axes of the Principal Component Analysis. (A) Points location reflects leaf quantitative traits variability. (B-F) Highlight the possible relationship with environmental/ecological variables. The PCA was made on Dataset 4 without LM_A data imputation. For details, see Material & Methods.

Table 1 Principal Component Analysis. Contribution of the different variables used in PCA to specimen ordination.

	PC1		PC2	
	imputation	no-imputation	imputation	no-imputation
All	54.2	56.1	29.7	29.0
Leaf size (MAX_Area)	33.1	32.2	1.2	0.4
Leaf width (MAX_width)	28.7	26.9	6.0	8.3
Leaf length (MAX_length)	16.4	20.5	34.8	26.8
Leaf l/w ratio (MAX_lw)	3.7	1.4	57.6	64.1
LM_A	18.1	19.0	0.4	0.4

Table 2 Results of Type-II ANOVA. ANOVAs were calculated on GLMs, including calculated LM_A (Dataset 4, n = 228 leaves). Chi-square values are displayed only for at least marginally significant variables, along with p-values. Some variables were excluded from the modeling (sometimes to allow the model to converge) and are marked as "not tested."

	Leaf traits				Plant-insect interaction		
	(SI-M1) LM_A		(SI-M2) Leaf size		Presence/absence	Damaged area	Herbivory index
	Chi2	p-value	Chi2	p-value	no model found		
LM_A	not tested		not tested				
Leaf size	48.52	< 0.001	not tested				
Phenology	68.89	< 0.001	11.46	< 0.01			
Locality	2.07	0.15	ns	ns			
Family	ns	ns	ns	ns			
Species	70.97	< 0.001	151.72	< 0.001			
Categorical TCT	ns	ns	ns	ns			
Combined TCT	ns	ns	ns	ns			
Growth form	ns	ns	ns	ns			
IA	ns	ns	18.42	< 0.001			
AIC	1930.31		3334.94				
R2	0.58		0.66				
n	228		228		228	18	18

Notes.

Chi2:	Chi-square values
LM_A:	Leaf mass per area
SI-M:	Supplemental Information-Model number (the formula in the text above)
Categorial TCT:	Classes of TCTs (TCT without considering the secondary venation type, e.g., EF)
Combined TCT:	TCTs based on a combined specimen and taxonomy-based approach; see Material & Methods
IA:	Preservation (Area) index (i.e., the proportion of a leaf preserved as fossil; see Material & Methods)
AIC:	Akaike Information Criterion
R2:	Nagelkerke's R2 (reflects the explanatory power of the models; function R2, package *performance*)
n:	Number of leaves used to fit the model's
ns:	"Nonsignificant" indicates variables whose effect was tested but which are not significant (see model selection procedure with the step function, see Material & Methods)
