# Supplementary material for: An integrated leaf trait analysis of two Paleogene leaf floras
Source: PeerJ. 2023 Apr 10;11:e15140. doi: 10.7717/peerj.15140 (PMC10100813; doi:10.7717/peerj.15140)
Supplement: Supplemental Information 3 — Additional discussion regarding the effect of potential biases in the fossil leaf and insect damage type (DT) record on the methods used. [file peerj-11-15140-s003.pdf]

Supplemental Article S3

Additional discussion is presented regarding the effect of potential biases in the fossil leaf and insect damage type (DT) record on the methods used.

Biases in the fossil leaf and insect damage type record

Studying fossil leaf assemblages (e.g., their taxonomic composition, insect herbivory pattern, TCT composition, and quantitative leaf traits), one must keep in mind the taphonomic characteristics of the assemblages, i.e., if the taphocoenosis are parautochthonous (i.e., assemblage derived from the surrounding vegetation) or allochthonous (i.e., assemblages with plant remains derived from a larger source area and potentially from several plant communities (e.g., *Kovar-Eder, 2016*)) by origin (*Behrensmeyer & Hook, 1992*). The leaf assemblages of Seifhennersdorf and Suletice-Berand have been described as parautochthonous taphocoenosis and are considered to document the local environment (*Kvaček & Walther, 1995*; *Walther & Kvaček, 2007*). The following sections discuss open questions and methodological issues that should be considered when dealing with herbivory patterns and leaf traits derived from fossil leaf assemblages to perform Integrated Leaf Trait Analysis.

Trait Combination Types

The fragmented habit of fossil leaves and sometimes difficult determination of specific morphological leaf traits, such as type of secondary venation, can cause an underrepresentation of TCTs in a dataset by specimen-based TCT analysis (*Roth-Nebelsick et al., 2017*). Therefore, a control based on published fossil-species lists, i.e., taxonomy-based TCT analysis (*Kunzmann et al., 2019*), should be additionally applied to record leaf morphotypes that are scarcely present and not well-preserved. Furthermore, the taxonomy-based TCTs can include specimens in a dataset that are taxonomically determinable but lack a morphological trait by preservation. The present study demonstrates that a combined specimen- and taxonomy-based TCT approach works well to record the diversity and abundance of morphotypes for whole leaf assemblages.

Leaf quantitative traits

A reliable acquisition of quantitative leaf traits highly depends on preserving leaf laminae, including petioles (*Royer et al., 2007*; *Traiser et al., 2018*). Leaf laminae should be preserved as completely as possible. *Moraweck et al. (2019)* recommended that a minimum of 70% (IA = 0.7) leaf surface area for a fossil leaf is a reasonably lower limit. Preservation is critical for LM_A calculations because it requires the measurement of petiole width at the point of attachment to the lamina *Royer et al. (2007)*. Regarding various preservation states or degrees of fragmentation of fossil leaves, the number of suitable specimens can considerably vary between entire assemblages and individual fossil-species within an assemblage. So far, sample sizes and sampling procedures in determining quantitative traits from fossil leaf assemblages are variable between studies (i.e., 90-250 leaves per site, *Roth-Nebelsick et al., 2017*; *Kunzmann et al., 2019*) and a standardized methodology is still pending.

The completeness of the leaf laminae and, thus, the lamina reconstruction depends on the size of the hand specimen, which is related to the cleavability of the rocks and the sampling techniques. The results of this (i.e., the negative correlation of leaf size and AI (PI) in GLM analysis) and previous studies (e.g., *Greenwood, 1992*; *Hagen et al., 2019*) indicate that large leaves (e.g., of *Dombeyopsis*) generally less likely to be completely preserved. Consequently, this leads to a possible underrepresentation of larger leaf sizes in the data. In contrast, fossil-species with small leaves (mean leaf area ~ 300-1000 mm²), such as Fabaceae div. sp., *Carpinus grandis*, *Zelkova zelkovifolia*, *Rosa lignitum*, and robust leaves, such as *Oleinites maii* or *Platanus neptuni*, can become more frequent in subsamples compared to their frequency in the entire assemblage. In addition, fossil-species with compound leaves (e.g., *Carya fragiliformis*, *Engelhardia orsbergensis*, or *Rosa lignitum*) are recorded mainly by disarticulated leaflets, resulting in higher frequencies being reported and traits that are related to the leaflet.

This unequal recording of different leaf types is particularly problematic if random subsamples from leaf collections are used for analyses without pre-assessing the quality and quantity of leaves for each fossil-species (see Material & Methods). With control, the validity of subsamples can be evaluated. The qualitative control indicates that the 400-specimen subsamples of both assemblages sufficiently represent the floristic composition of the entire collections with only occasional losses of very rare fossil-species (e.g., *Pungiphyllum cruciatum* in Suletice-Berand and *Oleinites hallbaueri* in Seifhennersdorf). As expected, the frequent fossil-species of the assemblages, such as *C. grandis*, *C. fragiliformis,* or *E. orsbergensis,* are also most frequently represented in the random samples. However, whether the frequency of fossil-species in a leaf assemblage represents, their original abundance in an ancient plant community is difficult to say. Based on the above discussions, data on quantitative traits should be considered cautiously for fossil leaf assemblages.

Insect herbivory

Damage frequency and DT richness determined for the Seifhennersdorf assemblage are very low compared to other Oligocene assemblages from Central Europe (*Wappler, 2010*; *Gunkel & Wappler, 2015*), despite the high amount of analyzed leaves. Many leaves must be sampled to identify diverse and rare DTs. It is also remarkable that multiple damaged leaves are rare (e.g., leaves showing more than one DT).

It remains an open question if the Seifhennersdorf result indicates a comparatively low intensity of insect herbivory in the paleo-ecosystem or if DTs are biased because of taphonomically more frequent preservation of undamaged leaves or the chipping of coalified material (see Material & Methods). To date, the effect of intensive herbivory on leaf preservation has been poorly studied. Two processes could be considered as being selective: (1) damaged leaves are only able to be transported for short distances from their mother plants and do not reach the potential fossil trap (physical selection), and (2) damaged leaves undergo biological decay more rapidly and are thus scarcely buried (biological selection).

References

Behrensmeyer AK, Hook RW. 1992. Paleoenvironmental contexts and taphonomic modes. In: Behrensmeyer A K Damuth J, DiMichele WA, Potts R, Sues H-D, Wing SL eds. *Terrestrial Ecosystems through Time*. Chicago: University of Chicago Press, 15–136.

Greenwood DR. 1992. Taphonomic constraints on foliar physiognomie interpretations of Late Cretaceous and tertiary palaeoeclimates. *Review of Palaeobotany and Palynology* 71:149–190 DOI 10.1016/0034-6667(92)90161-9.

Gunkel S, Wappler T. 2015. Plant-insect interactions in the upper Oligocene of Enspel (Westerwald, Germany), including an extended mathematical framework for rarefaction. *Palaeobiodiversity and Palaeoenvironments* 95:55–75 DOI 10.1007/s12549-014-0176-6.

Hagen ER, Royer DL, Moye RA, Johnson KR. 2019. No large bias within species between the reconstructed areas of complete and fragmented fossil leaves. *PALAIOS* 34:43–48 DOI 10.2110/palo.2018.091.

Kovar-Eder J. 2016. Early Oligocene plant diversity along the Upper Rhine Graben: the fossil flora of Rauenberg, Germany. *Acta Palaeobotanica* 56:329–440 DOI 10.1515/acpa-2016-0011.

Kunzmann L, Moraweck K, Müller C, Schröder I, Wappler T, Grein M, Roth-Nebelsick A. 2019. A Paleogene leaf flora (Profen, Sachsen-Anhalt, Germany) and its potentials for palaeoecological and palaeoclimate reconstructions. *Flora* 254:71–87 DOI 10.1016/j.flora.2018.11.005.

Kvaček Z, Walther H. 1995. The Oligocene volcanic flora of Suletice-Berand near Ústí nad Labem, North Bohemia–a review. *Fossil Imprint/Acta Musei Nationalis Pragae, Series B – Historia Naturalis Praha* 50:25–54.

Moraweck K, Grein M, Konrad W, Kvaček J, Kova-Eder J, Neinhuis C, Traiser C, Kunzmann L. 2019. Leaf traits of long-ranging Paleogene species and their relationship with depositional facies, climate and atmospheric CO2 level. *Palaeontographica Abteilung B* 298:93–172 DOI 10.1127/palb/2019/0062.

Roth-Nebelsick A, Grein M, Traiser C, Moraweck K, Kunzmann L, Kovar-Eder J, Kvaček J, Stiller S, Neinhuis C. 2017. Functional leaf traits and leaf economics in the Paleogene — A case study for Central Europe. *Palaeogeography, Palaeoclimatology, Palaeoecology* 472:1–14 DOI 10.1016/j.palaeo.2017.02.008.

Royer DL, Sack L, Wilf P, Lusk CH, Jordan GJ, Niinemets Ü, Wright IJ, Westoby M, Cariglino B, Coley PD, Cutter AD, Johnson KR, Labandeira CC, Moles AT, Palmer MB, Valladares F. 2007. Fossil leaf economics quantified: calibration, Eocene case study, and implications. *Paleobiology* 33:574–589 DOI 10.1666/07001.1.

Traiser C, Roth-Nebelsick A, Grein M, Kovar-Eder J, Kunzmann L, Moraweck K, Lange J, Kvaček J, Neinhuis C, Folie A, De Franceschi D, Kroh A, Prestianni C, Poschmann M, Wuttke M. 2018. MORPHYLL: A database of fossil leaves and their morphological traits. *Palaeontologia Electronica* 21:1–17 DOI 10.26879/773.

Walther H, Kvaček Z. 2007. Early Oligocene flora of Seifhennersdorf (Saxony). *Fossil Imprint/Acta Musei Nationalis Pragae, Series B–Historia Naturalis* 63:85–174.

Wappler T. 2010. Insect herbivory close to the Oligocene–Miocene transition – A quantitative analysis. *Palaeogeography, Palaeoclimatology, Palaeoecology* 292:540–550 DOI 10.1016/j.palaeo.2010.04.029.
