# Supplementary figures and images for: An integrated leaf trait analysis of two Paleogene leaf floras
Source: PeerJ. 2023 Apr 10;11:e15140. doi: 10.7717/peerj.15140 (PMC10100813; doi:10.7717/peerj.15140)

**A**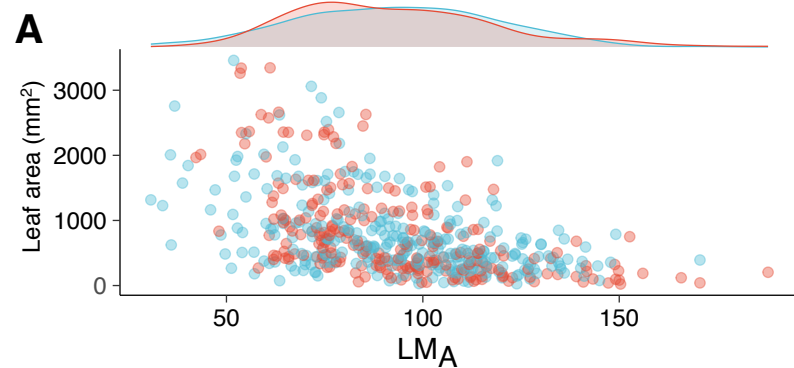**B**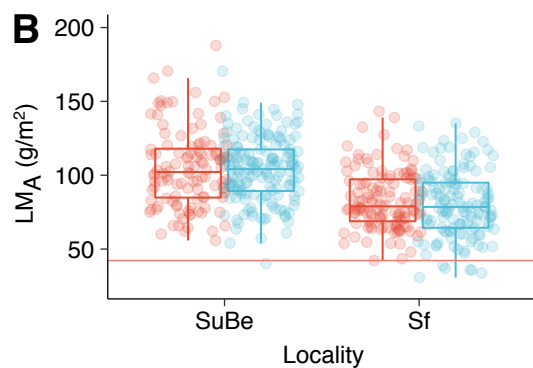**C**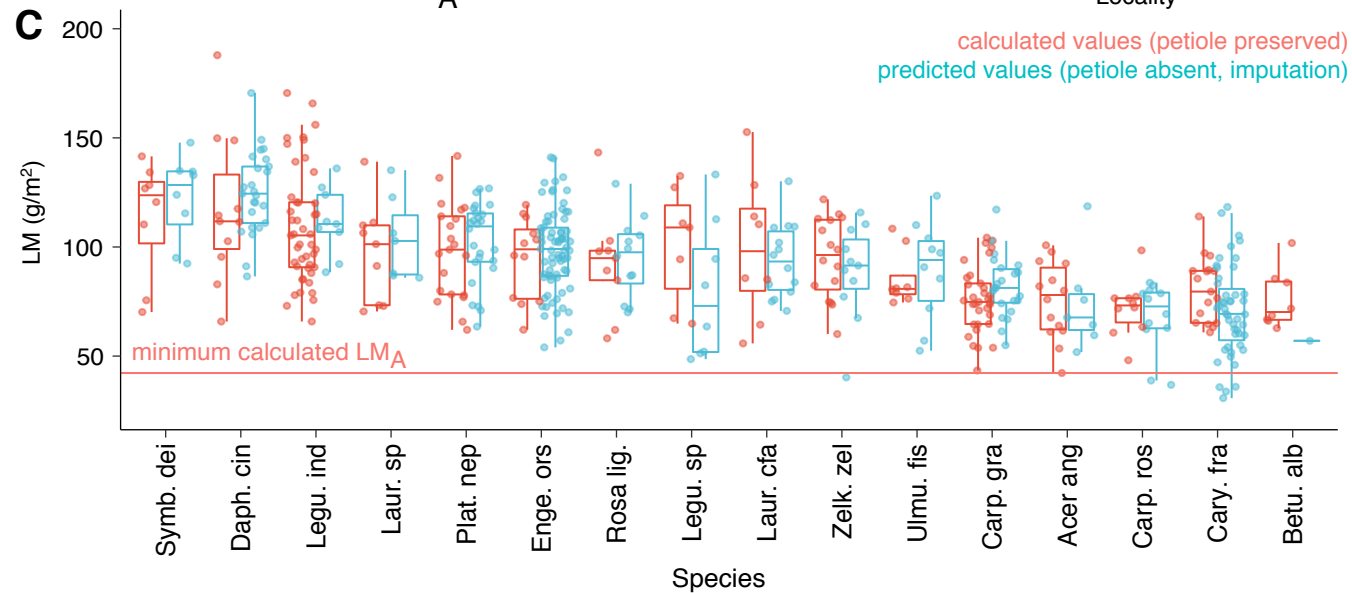

Supplement: Supplemental Information 11 — There are two types of LMA: calculated LMA shown in blue and predicted (simulated) LMA in red. Each imputed value (data point) averages 10 imputation cycles per specimen. (A) The relationship between LMA and leaf area is shown for both types of LMA. Above are the associated density curves. (B) Compared are both types of LMA per locality. (C) Compared are both types of LMA per fossil-species. Five simulated LMA values are below the range of the calculated LMA. They were removed from the multivariate analyses. See Material & Methods for further information. [file peerj-11-15140-s011.pdf]
