# Supplementary material for: An integrated leaf trait analysis of two Paleogene leaf floras
Source: PeerJ. 2023 Apr 10;11:e15140. doi: 10.7717/peerj.15140 (PMC10100813; doi:10.7717/peerj.15140)
Supplement: Supplemental Information 15 [file peerj-11-15140-s015.pdf]

Taxonomy	n	n (LM_A)	Mean LM_A (g/m^2)	Pheno	TCT	% Dam	DT list	DTO	DT richness
**Betulaceae**	1691					5.26	1;2;3;5;12;14;16;26;57;78;81;214	99	1.04 (SD = 0.97)
Carpinus grandis	1068	36	75.25 (SD = 14.88)	d	F	4.40	2;3;5;12;14;16;26;57;78;81;214	50	0.86 (SD = 0.87)
Carpinus roscheri	284	10	71.91 (SD = 12.44)	d	F	8.45	1;2;3;5;12;14;57;78;214	29	1.77 (SD = 1.25)
Betula alboides	66	9	76.09 (SD = 11.78)	d	F	9.09	2;14;57	7	1.47 (SD = 0.79)
**Juglandaceae**	1293					6.65	1;2;3;5;12;13;14;15;16;50;57;81	99	1.30 (SD = 1.08)
Carya fragiliformis	1262	17	80.49 (SD = 14.42)	d	F	6.81	1;2;3;5;12;13;14;15;16;50;57;81	99	1.33 (SD = 1.09)
Cyclocarya sp.	22			d	F				
**Lauraceae**	470					3.62	2;12;14;30;31;81	17	0.68 (SD = 0.75)
D. cinnamomifolia	103	7	125.65 (SD = 21.92)	e	C, D	1.94	31;81	2	0.39 (SD = 0.56)
L. acutimontanum	31			e	A	9.68	2;12;14	3	2.00 (SD = 0.79)
**Sapindaceae**	421					2.38	2;5;12;14;32;57	15	0.67 (SD = 1.00)
A. (cf.) *angustilobum*	140	15	75.20 (SD = 16.54)	d	P	1.43	12;32	2	0.29 (SD = 0.49)
A. (cf.) *tricuspidatum*	73			d	P	2.74	2;5;12	4	1.04 (SD = 1.28)
A. rueminianum	32	5	84.70 (SD = 29.76)	d	P	6.25	2	2	0.88 (SD = 0.32)
A. palaeosaccharinum	21			d	P	4.76	5;57	2	2.00 (SD = 0.00)
**Rosaceae**	251					2.79	12;14	7	0.50 (SD = 0.59)
Rosa lignitum	248	9	92.39 (SD = 23.43)	d	E	2.82	12;14	7	0.50 (SD = 0.59)
**Ulmaceae**	183					2.19	12;13	4	0.41 (SD = 0.55)
Ulmus fischeri	115	8	85.54 (SD = 11.88)	d	F	2.61	12;13	3	0.50 (SD = 0.60)
Zelkova zelkovifolia	30			d	F				
**Platanaceae** ≙ *Platanus neptuni*	82	10	108.93 (SD = 24.59)	d	E	6.10	2;12;14	5	1.12 (SD = 0.81)
**Magnoliaceae** ≙ *M. seifhennersdorfensis*	64			e	A, B	1.56	2	1	0.32 (SD = 0.47)
**Salicaceae**	39					5.13	5;15	2	1.05 (SD = 0.70)
Salix varians	23			d	F	4.35	15	1	0.91 (SD = 0.29)
**Fagaceae**	33					6.06	2;12	2	1.25 (SD = 0.67)
C. lonchitiforme	24			e	F	4.17	2	1	0.87 (SD = 0.34)
**Simaroubaceae** ≙ *Ailanthus prescheri*	29			d	E, F				

Notes.

n:	Number of leaves in the assemblage
n (LM_A):	Number of leaf mass per area (LM_A) values
Pheno:	Phenology (d: deciduous, e: evergreen, see Material & Methods)
TCT:	Taxonomy-based Trait Combination Type (see Material & Methods)
% DAM:	Percentage of damaged leaves (damage frequency, see Material & Methods)
DT list:	List of insect damage types (DTs) after Labandeira et al. (2007)
DTO:	Damage type occurrence (see Material & Methods)
DT richness:	Damage type richness standardized on 20 leaves (see Material & Methods)
SD:	Standard deviation
A.:	*Acer*
C.:	*Castaneophyllum*
D.:	*Daphnogene*
L.:	*Laurophyllum*
M.:	*Magnolia*
