# Supplementary material for: An integrated leaf trait analysis of two Paleogene leaf floras
Source: PeerJ. 2023 Apr 10;11:e15140. doi: 10.7717/peerj.15140 (PMC10100813; doi:10.7717/peerj.15140)
Supplement: Supplemental Information 16 [file peerj-11-15140-s016.pdf]

**Taxonomy**	**n**	**n (LM_A)**	**Mean LM_A (g/m^2)**	**Pheno**	**TCT**	**% Dam**	**DT list**	**DTO**	**DT richness**
**Juglandaceae**	404					12.62	1;2;3;5;7;12;13;14;16;17;26;30;50;61	56	2.25 (SD = 1.25)
E.[10] *orsbergensis*	380	12	94.91 (SD = 17.94)	e	E	11.84	1;2;3;5;7;12;13;14;16;30;61	49	2.03 (SD = 1.14)
**Lauraceae**	361					10.80	1;2;3;4;5;7;12;13;14;20;61;127	46	2.22 (SD = 1.41)
D.[11] *cinnamomifolia*	92	11	117.15 (SD = 32.52)	e	C, D	8.70	1;3;5;12;13;14	10	1.94 (SD = 1.23)
L.[12] cf. *acutimontanum*	77	8	99.55 (SD = 30.67)	e	A	10.39	2;3;4;12;13;14;20	11	2.58 (SD = 1.47)
L.[12] cf. *pseudoprinceps*	50			e	A	4.00	12	2	0.65 (SD = 0.48)
**Elaeocarpaceae ≙**<br>*Sloanea olmediifolia*	112			e	F	20.54	2;3;5;12;17;20;30;57;61	26	3.30 (SD = 1.25)
**Platanaceae ≙**<br>*Platanus neptuni*	99	12	95.10 (SD = 23.49)	d	E	8.08	2;5;12;16;18;38	11	1.98 (SD = 1.31)
**Sapindaceae**	70					17.14	1;2;3;5;12;50	15	3.20 (SD = 1.45)
Acer tricuspidatum	21			d	P	28.57	1;2;3;50	8	4.00 (SD = 0.00)
**Ulmaceae ≙**<br>*Zelkova zelkovifolia*	55	13	99.24 (SD = 15.64)	d	F	12.73	2;3;5;12;16;78	9	2.85 (SD = 1.23)
**Symplocaceae ≙**<br>*S.*[13] *deichmuelleri*	29	8	113.45 (SD = 28.93)	e	E, F	3.45	13	1	0.71 (SD = 0.45)
**Magnoliaceae ≙**<br>*Magnolia* sp.	22			e	A				
**Cornaceae ≙**<br>*Cornus studeri*	20			d	B	5.00	1	1	1.00 (SD = 0.00)

Notes.

n: Number of leaves in the assemblage

n (LM_A): Number of leaf mass per area (LM_A) values

Pheno: Phenology (d: deciduous, e: evergreen, see Material & Methods)

TCT: Taxonomy-based Trait Combination Type (see Material & Methods)

% DAM: Percentage of damaged leaves (damage frequency, see Material & Methods)

DT list: List of insect damage types (DTs) after Labandeira et al. (2007)

DTO: Damage type occurrence (see Material & Methods)

DT richness: Damage type richness standardized on 20 leaves (see Material & Methods)

SD: Standard deviation

D.: *Daphnogene*

E.: *Engelhardia*

L.: *Laurophyllum*

S.: *Symplocos*
