# Supplementary material for: An integrated leaf trait analysis of two Paleogene leaf floras
Source: PeerJ. 2023 Apr 10;11:e15140. doi: 10.7717/peerj.15140 (PMC10100813; doi:10.7717/peerj.15140)
Supplement: Supplemental Information 17 — ‘ANOVAs were calculated on GLMs, including calculated and simulated LMA (Dataset 5, n = 506 leaves). Chi-square values are displayed only for at least marginally significant variables, along with p-values. Some variables were excluded from the modeling (sometimes to allow the model to converge) and are marked as “not tested”’. [file peerj-11-15140-s017.pdf]

	Leaf traits				Plant-insect interaction					
	(M1) LM_A		(M2) Leaf size		(M3) Presence/absence		(M4) Damaged area		(M5) Herbivory index	
	Chi2	p-value	Chi2	p-value	Chi2	p-value	Chi2	p-value	Chi2	p-value
LM_A	not tested		not tested		4.97	< 0.001	ns	ns	2.46	0.12
Leaf size	71.64	< 0.001	not tested		2.60	0.11	9.94	< 0.01	not tested	
Phenology	56.17	< 0.001	13.41	< 0.01	ns	ns	10.85	< 0.01	8.64	0.01
Locality	2.14	0.14	2.89	0.09	13.39	< 0.001	ns	ns	ns	ns
Family	ns	ns	ns	ns	ns	ns	ns	ns	ns	ns
Species	146.73	< 0.001	149.38	< 0.001	ns	ns	ns	ns	ns	ns
Categorical TCT	ns	ns	ns	ns	8.41	0.04	ns	ns	ns	ns
Combined TCT	ns	ns	ns	ns	ns	ns	ns	ns	ns	ns
Growth form	ns	ns	ns	ns	ns	ns	ns	ns	ns	ns
IA	6.18	0.01	18.09	< 0.001	ns	ns	not tested		not tested	
AIC	4431.38		7525.76		315.97		426.01		236.26	
R2	0.52		0.53		0.06		0.58		0.24	
n	506		506		506		50		50	

Notes.

Chi2:	Chi-square values
LM_A:	Leaf mass per area
M:	Model number (formula given in Results)
Categorial TCT:	Classes of TCTs (TCT without considering the secondary venation type, e.g., EF)
Combined TCT:	TCTs based on a combined specimen and taxonomy-based approach; see Material & Methods
IA:	Preservation (Area) index (i.e., the proportion of a leaf preserved as fossil; see Material & Methods)
AIC:	Akaike Information Criterion
R2:	Nagelkerke's R2 (reflects the explanatory power of the models; function R2, package *performance*)
n:	Number of leaves used to fit the model’s
ns:	“Nonsignificant” indicates variables whose effect was tested but which are not significant (see model selection procedure with the step function, see Material & Methods)
